# In vitro immune responses of porcine alveolar macrophages reflect host immune responses against porcine reproductive and respiratory syndrome viruses

**DOI:** 10.1186/s12917-018-1675-x

**Published:** 2018-12-04

**Authors:** Nadeem Shabir, Amina Khatun, Salik Nazki, Suna Gu, Sang-Myoung Lee, Tai-Young Hur, Myoun-Sik Yang, Bumseok Kim, Won-Il Kim

**Affiliations:** 1 College of Veterinary Medicine, Chonbuk National University, 79 Gobong-ro, Iksan, Jeonbuk Korea; 2 Division of Animal Biotechnology, Faculty of Veterinary Sciences and Animal Husbandry, Sher-e-Kashmir University of Agricultural Sciences and Technology of Kashmir, Srinagar, India; 3 College of Environmental & Biosource Science, Division of Biotechnology, Chonbuk National University, Iksan, South Korea; 4 Dairy Science Division, National Institute of Animal Science, Rural Development Administration, Cheonan, 31000 South Korea

**Keywords:** PRRSV, Immune response, Alveolar macrophages, Peripheral blood mononuclear cells, Flow cytometry

## Abstract

**Background:**

Currently, an in vitro immunogenicity screening system for the immunological assessment of potential porcine reproductive and respiratory syndrome virus (PRRSV) vaccine candidates is highly desired. Thus, in the present study, two genetically divergent PRRSVs were characterized in vitro and in vivo to identify an in vitro system and immunological markers that predict the host immune response. Porcine alveolar macrophages (PAMs) and peripheral blood mononuclear cells (PBMCs) collected from PRRSV-negative pigs were used for in vitro immunological evaluation, and the response of these cells to VR2332c or JA142c were compared with those elicited in pigs challenged with the same viruses.

**Results:**

Compared with VR2332c or mock infection, JA142c induced increased levels of type I interferons and pro-inflammatory cytokines (TNF-α, IL-1α/β, IL-6, IL-8, and IL-12) in PAMs, and these elevated levels were comparable to the cytokine induction observed in PRRSV-challenged pigs. Furthermore, significantly greater numbers of activated CD4^+^ T cells, type I helper T cells, cytotoxic T cells and total IFN-γ^+^ cells were observed in JA142c-challenged pigs than in VR2332c- or mock-challenged pigs.

**Conclusions:**

Based on these results, the innate immune response patterns (particularly IFN-α, TNF-α and IL-12) to specific PRRSV strains in PAMs might reflect those elicited by the same viruses in pigs.

## Background

Porcine reproductive and respiratory syndrome virus (PRRSV), a positive-sense RNA virus belonging to the family *Arteriviridae* of the order *Nidovirales,* exerts a significant economic impact on the swine industry worldwide [1–3], with an estimated annual loss of at least $664 million in the USA alone [4]. PRRSV possesses a 15-kb polycistronic genome containing two large open reading frames (ORFs 1a and 1b) that encode non-structural proteins (NSPs) and eight structural protein-encoding ORFs (2a, 2b, 3, 4, 5a, 5, 6 and 7) [5, 6]. PRRSV is broadly classified into two divergent genotypes, European (Type I) and North American (Type II), with Type II PRRSVs further sub-divided into at least nine different genetic lineages with inter-lineage genetic distances varying from 11 to 18% [7, 8]. The high genetic variability that exists between different PRRSV strains has resulted in increased antigenic and immunological diversity [9, 10]. This diversity among PRRSVs not only hinders our understanding of PRRSV-induced immunity in pigs but also poses a serious hurdle for the selection of immunogenic strains for vaccine development [11]. Moreover, due to the lack of immunological classification of divergent PRRSV strains and reliable parameters for predicting vaccine protection, the immunogenicity and protective efficacy of PRRSV vaccine candidates can be only evaluated through challenge experiments in pigs [12].

The immune responses elicited by PRRSV in vitro and in vivo have been well-studied, but researchers have not reached a consensus on immune response patterns. PRRSV reportedly up-regulates IL-10 [13–15] and induces insignificant amounts of or inhibits the expression of type I interferons (IFNs) and TNF-α in vitro and/or in vivo [16–20]. However, some in vitro and in vivo studies have also demonstrated that the induction of these cytokines is strain dependent [21–24]. According to the majority of studies, PRRSVs induce IL-8 [25, 26], and the in vitro*/*ex vivo induction of IL-6 by PRRSV has also been reported [27–29].

Our current understanding of cell-mediated immune responses to PRRSV is unclear. IFN-γ has been used as a marker of Th1 polarization during PRRSV infection [30, 31]. Cytotoxic T cell activity against PRRSV-infected macrophages was only induced at 49 days post-infection [32], and weak PRRSV-specific cytotoxic T cell responses (CD8^+^IFN-γ^+^) were reported in another study [33]. Furthermore, PRRSV infection increases the number of TGF-β producing regulatory T cells (T_Regs_) [34]. In addition, monocyte-derived dendritic cells (MoDCs) infected with PRRSV do not increase the frequency and proliferation of T_Regs_ in an in vitro co-culture system [35]. The negligible innate immune responses induced by PRRSV may severely affect adaptive immunity; along with other irregular adaptive immune processes, this impaired response leads to overall immune inefficiency and co-infections [36]. Therefore, it is important to understand early innate immunity events to gain insight into the immune response to PRRSV infection [37, 38].

The role of neutralizing antibodies (NAbs) during PRRSV infection is not completely understood [39]. PRRSV NAbs usually appear during the third or fourth week post-infection [36], and an NAb titre of 1:8 is sufficient to block viremia in PRRSV-infected pigs. However, NAb induction may be dependent on the strain of PRRSV [40, 41].

Given the strain-dependent elicitation of innate, cell-mediated and humoral immune responses by PRRSV, multiple virus strains should be investigated to gain a better understanding of PRRSV immunobiology [9]. Thus, there is a need to select an in vitro system to immunologically classify diverse PRRSVs and to predict the protection and immune responses induced by vaccine candidates. The majority of previous studies examining PRRSV-induced immune responses have used a single PRRSV strain that was tested either in vitro or in vivo but rarely both. These studies also failed to take into account the effects of PRRSV strain diversity, immune evaluation systems, or both when drawing general conclusions regarding PRRSV immunobiology. To fill in these gaps, the current study focused on two important goals: i) in vitro and in vivo characterization of the immune responses elicited by two divergent Type II PRRSV strains, and ii) identification of a feasible in vitro model or specific immunological markers to predict the in vivo immune responses induced by a PRRSV strain.

## Results

### PRRSVs replicated at similar levels in PAMs and pigs

**Fig. 1:**
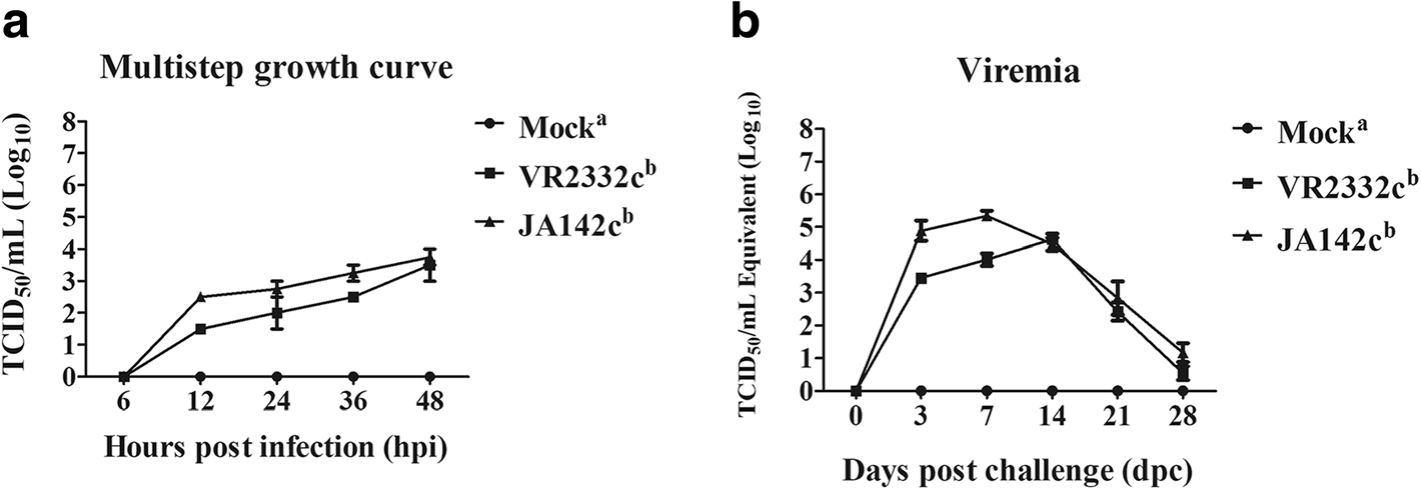
PRRSV replication in PAMs and pigs. **a** Multi-step growth curve of PRRSVs in PAMs (*n* = 6) at 6, 12, 24, 36 and 48 hpi, as determined by titration using MARC-145 cells. **b** Viral loads in pig sera (*n* = 5) at 0, 3, 7, 14, 21 and 28 dpc, as determined by a quantitative real-time PCR for PRRSV. The bars represent the means, and the error bars represent the standard errors of the mean (SEM). Bars showing different letters represent values that differ significantly from each other (*p* < 0.05)

A multi-step growth curve for PRRSV replication in PAMs was constructed by measuring viral titres in cell lysates at 6, 12, 24, 36 and 48 h post-infection (hpi). Slightly higher levels of viral replication were observed for JA142c than for VR2332c in PAMs (Fig. 1a) and in pigs (Fig. 1b), but these differences were not statistically significant. Neither strain produced viral titres at 6 hpi in PAMs, but JA142c and VR2332c exhibited titres of 10^2.5^ and 10^1.5^ TCID_50_/mL at 12 hpi, gradually increasing to 10^3.75^ and 10^3.5^ TCID_50_/mL, respectively, at 48 hpi. In a pig challenge model, the animals were tested for viremia at 0, 3, 7, 14, 21 and 28 dpc. The mean levels of viremia in pigs challenged with VR2332c or JA142c peaked at 14 and 7 dpc, with values of 10^4.6^ and 10^5.3^ TCID_50_/mL, and then gradually decreased to 10^0.5^ and 10^1.1^ log_10_TCID_50_/mL, respectively. Overall, JA142c and VR2332c demonstrated similar levels of replication in pigs and PAMs.

### JA142c exhibited increased cytokine transcript and protein levels in PAM and PBMC cultures

**Fig. 2:**
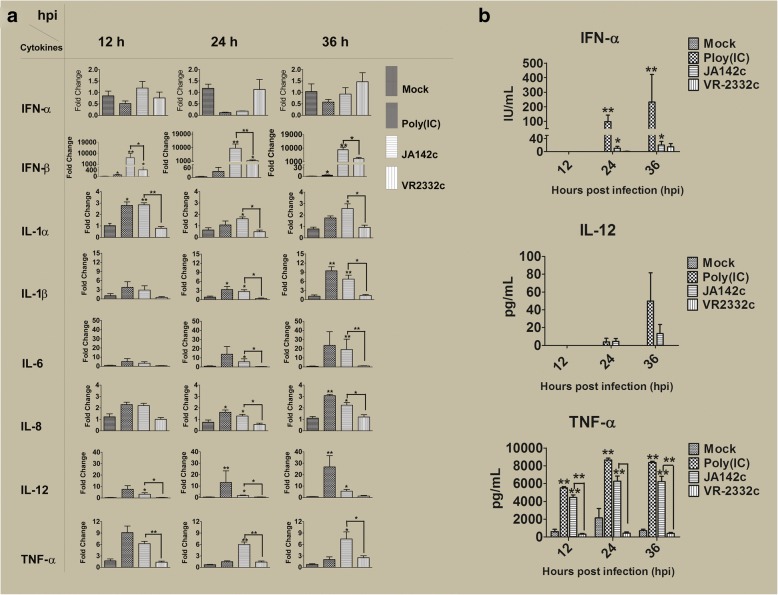
Cytokine expression in PAM cultures. **a** mRNA and **b** protein expression of cytokines in PAMs and supernatants (*n* = 6), as determined by real-time PCR and ELISA, respectively, at 12, 24 and 36 hpi. Asterisks indicate significant differences in the cytokine expression induced by each virus or stimulant compared with mock treatment or significant differences between JA142c and VR2332c (* indicates *p* ≤ 0.05, ** indicates *p* ≤ 0.01)

**Fig. 3:**
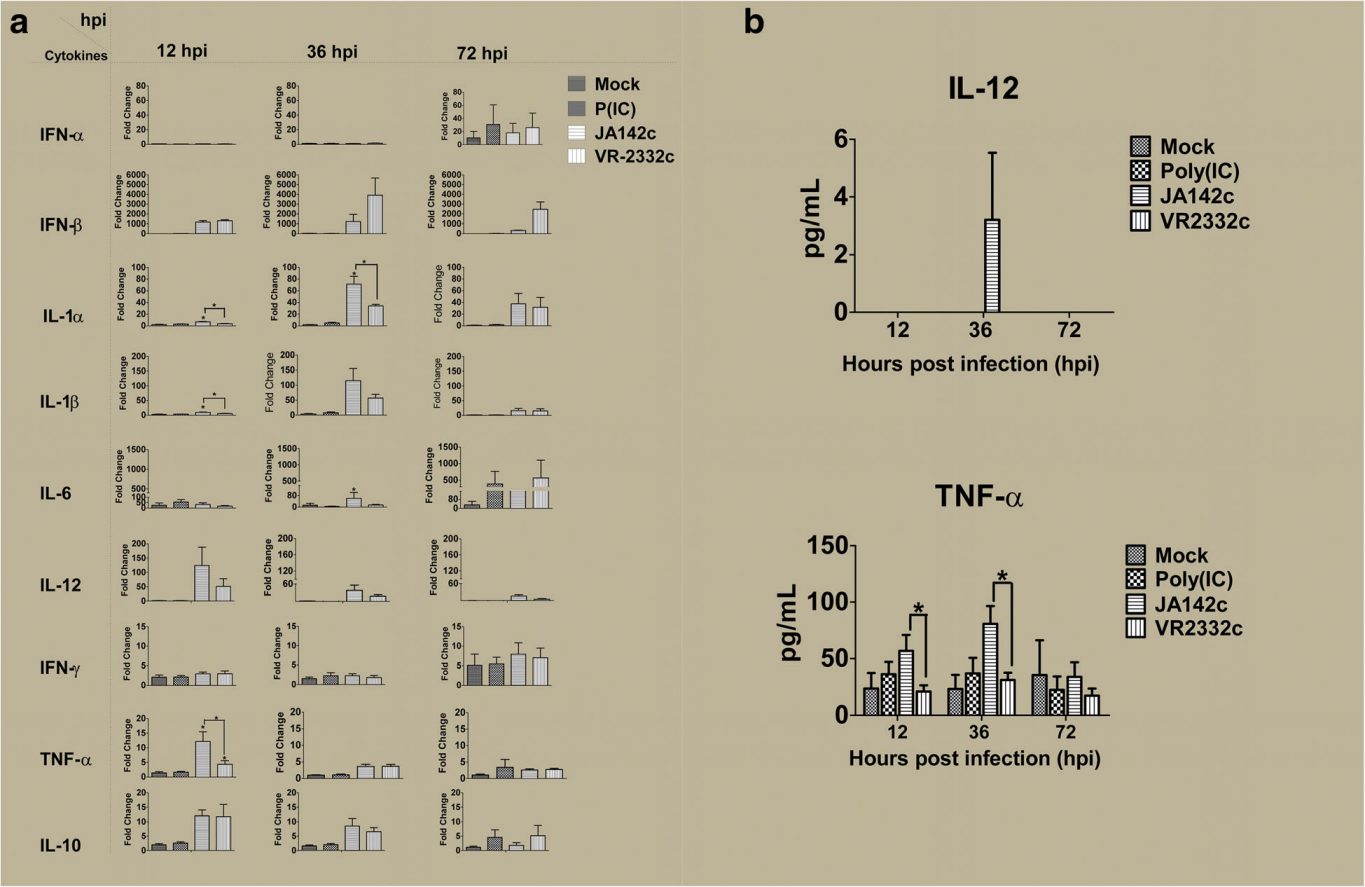
Cytokine expression in PBMC cultures. **a** mRNA and **b** protein expression of cytokines in PBMCs and supernatants (n = 6), as determined by real-time PCR and ELISA, respectively, at 12, 36 and 72 hpi. Asterisks indicate significant differences in cytokine expression induced by each virus or stimulant compared with mock treatment or significant differences between JA142c and VR2332c (* indicates *p* ≤ 0.05, ** indicates *p* ≤ 0.01)

Cytokine levels in PAMs, PBMCs and cell supernatants or lysates were evaluated by real-time PCR and/or ELISA. Compared to those of mock infection or VR2332c, JA142c and poly I:C induced significantly increased transcript levels of IFN-β and pro-inflammatory cytokines (IL-1α, IL-1β, IL-6, IL-8, IL-12, and TNF-α) at either two or all three time points assayed (12, 24 and 36 hpi) (Fig. 2a). Furthermore, significantly higher TNF-α and IFN-α levels were observed in the cell lysates of JA142c-infected PAMs than in the cell lysates of the mock- or VR2332c-infected groups for at least two out of three time points (Fig. 2b). However, there was no significant difference in IL-12 protein expression in PAM lysates between groups. In naïve PBMCs, JA142c induced significantly higher IL-1α, IL-1β and TNF-α mRNA transcript levels at 12 and/or 36 hpi (Fig. 3a). Further, TNF-α protein expression in JA142c-stimulated naive PBMCs was significantly higher at 12 and 36 hpi (Fig. 3b).

### JA142c induced higher levels of type I IFNs and pro-inflammatory cytokines in pigs

**Fig. 4:**
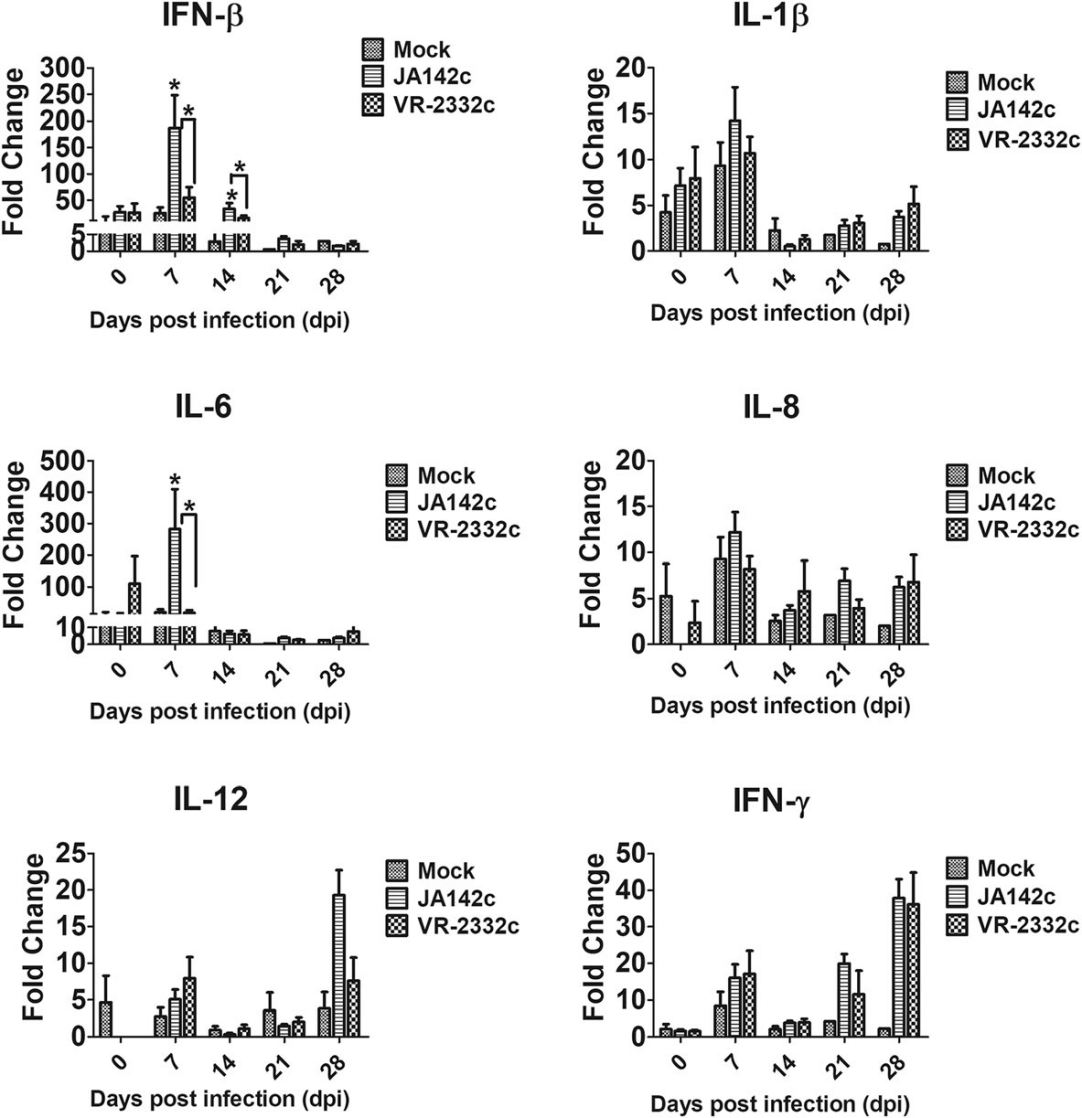
mRNA transcription of cytokines in PBMCs collected from pigs infected with the VR2332c and JA142c PRRSV strains at 0, 7, 14, 21 and 28 dpc. Analysis of mRNA transcription in PBMCs (n = 5) collected from infected and control pigs using real-time PCR. Asterisks indicate significant differences in cytokine expression induced by each virus compared with mock treatment or significant differences between JA142c and VR2332c (* indicates *p* ≤ 0.05)

**Fig. 5:**
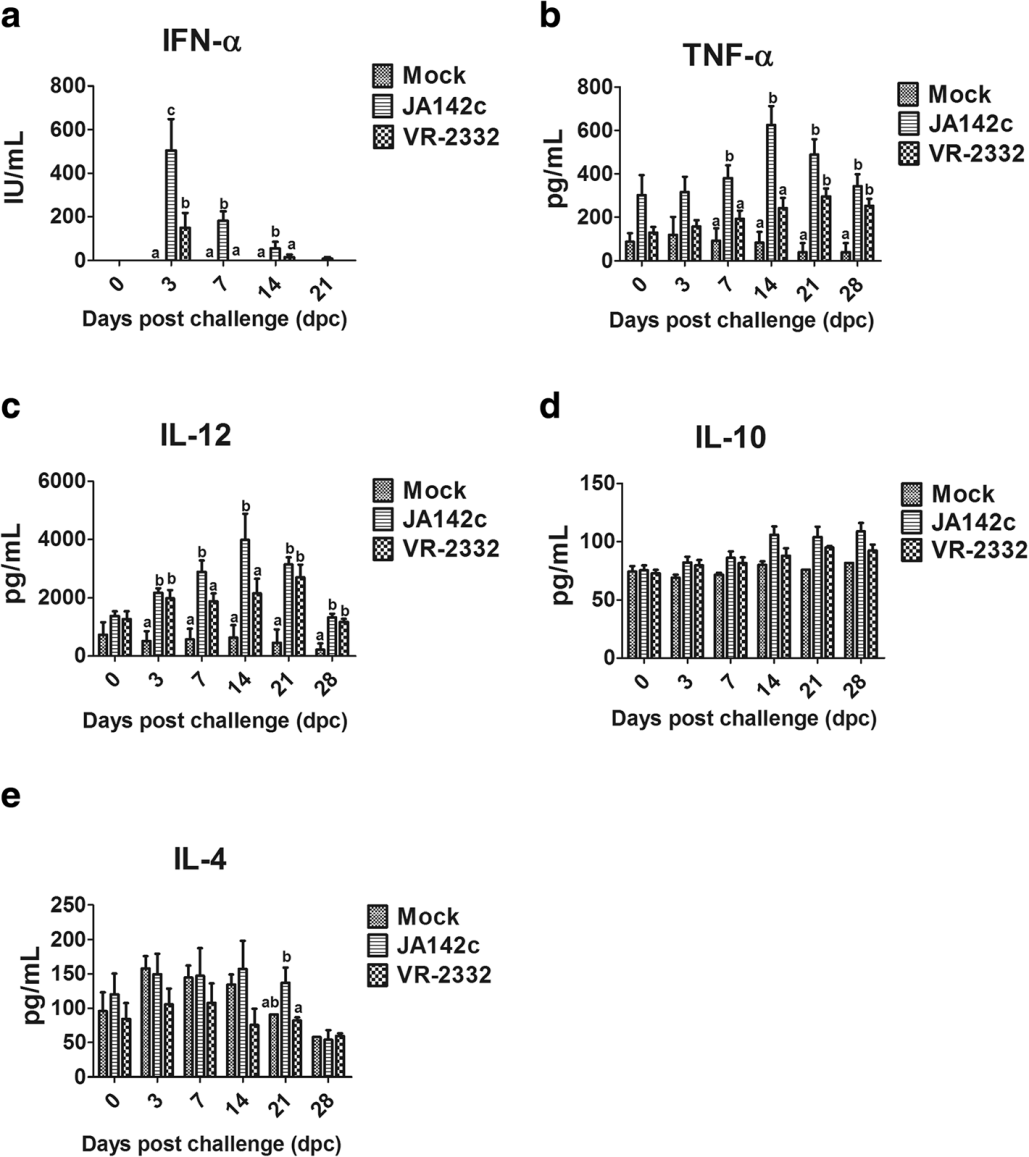
Serum cytokine levels in PRRSV-infected pigs. Sera collected from pigs (n = 5) at 0, 3, 7, 14, 21 and 28 dpc were analysed for **a** IFN-α; **b** TNF-α; **c** IL-12; **d** IL-10; and **e** IL-4 by ELISA. Each bar represents the average cytokine amount from 5 pigs ± SEM. Bars showing different letters represent values that differ significantly from each other (*p* < 0.05)

Regarding cytokine mRNA transcript levels in PBMCs collected from virus-challenged pigs, JA142c-infected pigs had significantly higher transcript levels of IFN-β and IL-6 that pigs subjected to other treatments at 7 or 14 dpc. The transcript levels of IL-8, IL-12 and IFN-γ at 7, 21 or 28 dpc were higher in JA142c-infected pigs than in the other groups, but these differences were not statistically significant (Fig. 4). Serum cytokines in pigs were evaluated by performing ELISA (Fig. 5). Although a significantly stronger induction of serum IFN-α was observed in VR2332c-infected pigs than in mock-infected pigs at 3 dpc, JA142c induced serum IFN-α levels that were significantly and consistently higher than those in either the VR2332c- or mock-infected groups until 14 dpc. Moreover, serum pro-inflammatory cytokine induction was enhanced. Specifically, TNF-α and IL-12 levels were significantly higher in JA142c-infected pigs from 7 to 14 dpc than in VR2332c- and mock-infected pigs; however, comparable levels of these cytokines were found in JA142c- and VR2332c-infected pigs at 21 and 28 dpi. JA142c also induced significantly higher serum levels of IL-4 at 21 dpc than did VR2332c.

**Fig. 6:**
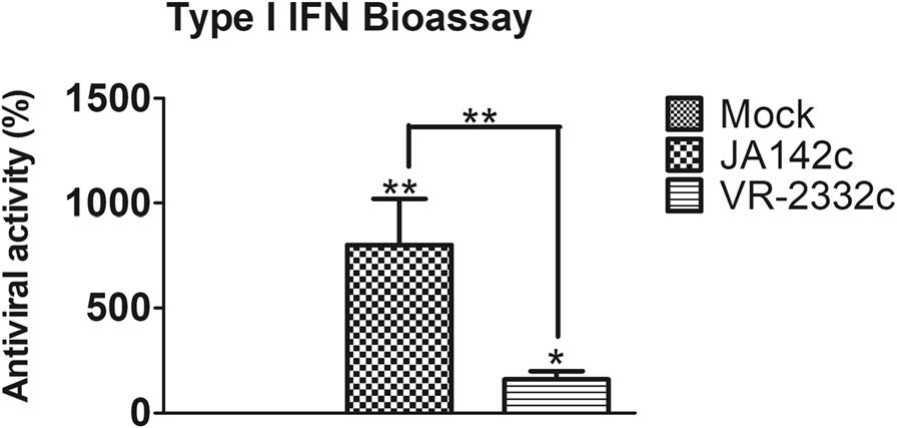
Type I IFN bioassay using sera from PRRSV-challenged pigs at 3 dpc. MDBK cells (n = 6) in 96-well plates were infected with VSV and incubated until a CPE was evident in mock-treated control cells. Type I IFN bioactivity in the serum was measured by performing a type I IFN bioassay

A type I IFN bioassay using sera from virus-challenged pigs at 3 dpc revealed significantly higher antiviral activity in JA142c-challenged pigs than in mock- and VR2332c-challenged pigs (Fig. 6).

### JA142c induced an enhanced cell-mediated immune response in pigs

**Fig. 7:**
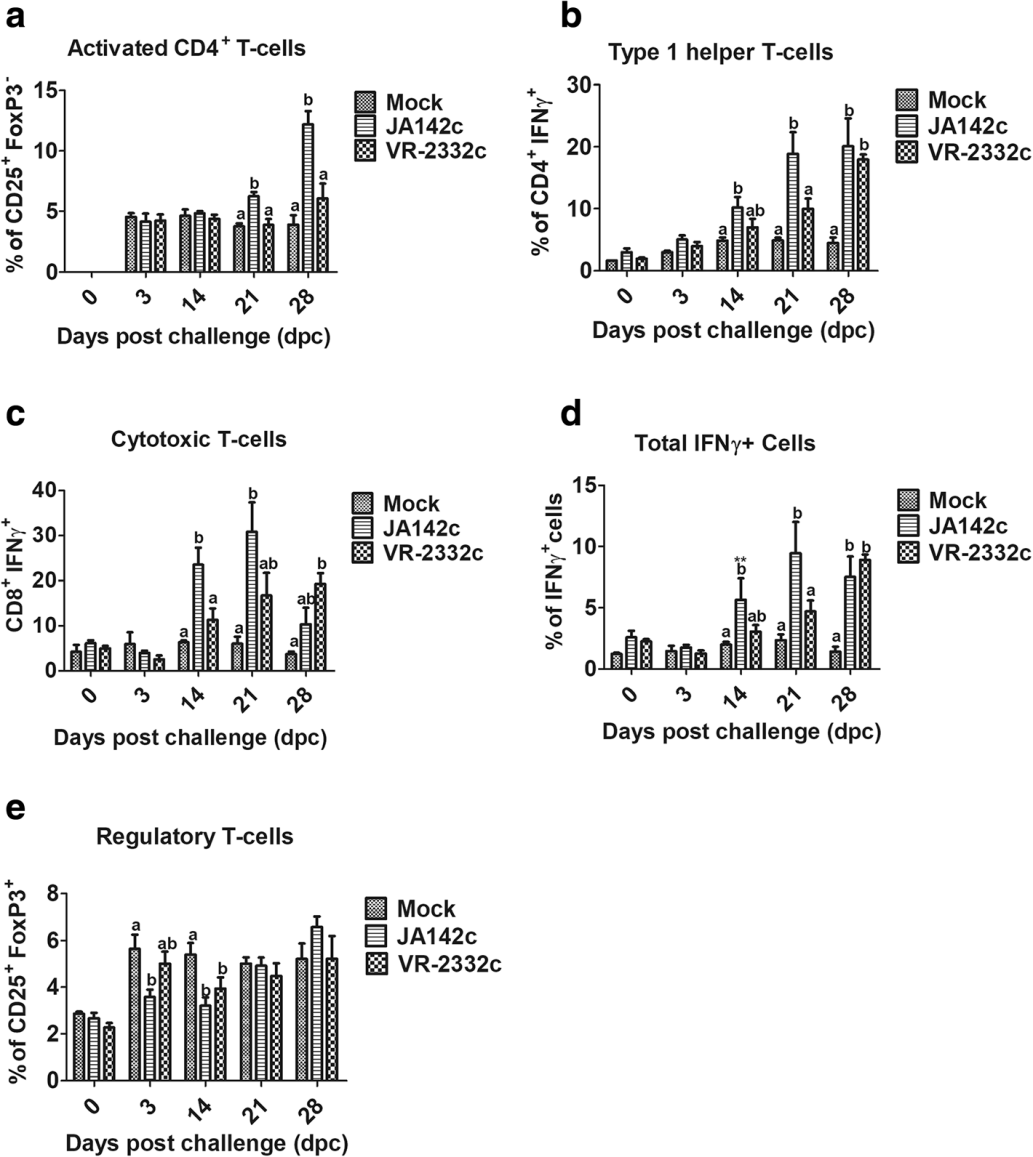
Frequency of immune cells in PBMCs derived from PRRSV-infected pigs. **a** Activated CD4^+^ T cells (CD4^+^CD25^+^Foxp3^−^), **b** type I helper T cells (CD4^+^IFN-γ^+^), **c** cytotoxic T cells (CD8^+^IFN-γ^+^), **d** total PBMCs expressing IFN-γ^+^ and **e** T_Regs_ (CD4^+^CD25^+^Foxp3^+^). Each bar represents the average percentage of immune cells from 5 pigs ± SEM. Bars showing different letters represent values that differ significantly from each other (*p* < 0.05)

As shown in Fig. 7, JA142c induced a significantly higher proportion of activated CD4^+^ T cells (CD4^+^CD25^+^FoxP3^−^) at 21 and 28 dpc than did mock or VR2332c challenge in pigs. In addition, significantly higher numbers of type I helper T cells (CD4^+^IFN-γ^+^) and cytotoxic T cells (CD8^+^IFN-γ^+^) among PBMCs were observed in JA142c-infected pigs at 14 and 21 dpc than in mock- or VR2332c-challenged pigs. Total IFN-γ^+^ lymphocytes in the JA142c-infected group were significantly higher at 14 and 21 dpc. Furthermore, regulatory T cell (CD4^+^CD25^+^FoxP3^+^) numbers were significantly lower in JA142c-infected pigs at 3 dpc and in JA142c- and VR2332c-infected pigs at 14 dpc than in the mock-infected group, although these levels gradually increased by 28 dpc in both groups.

### Induction of PRRSV-specific antibodies after challenge

**Fig. 8:**
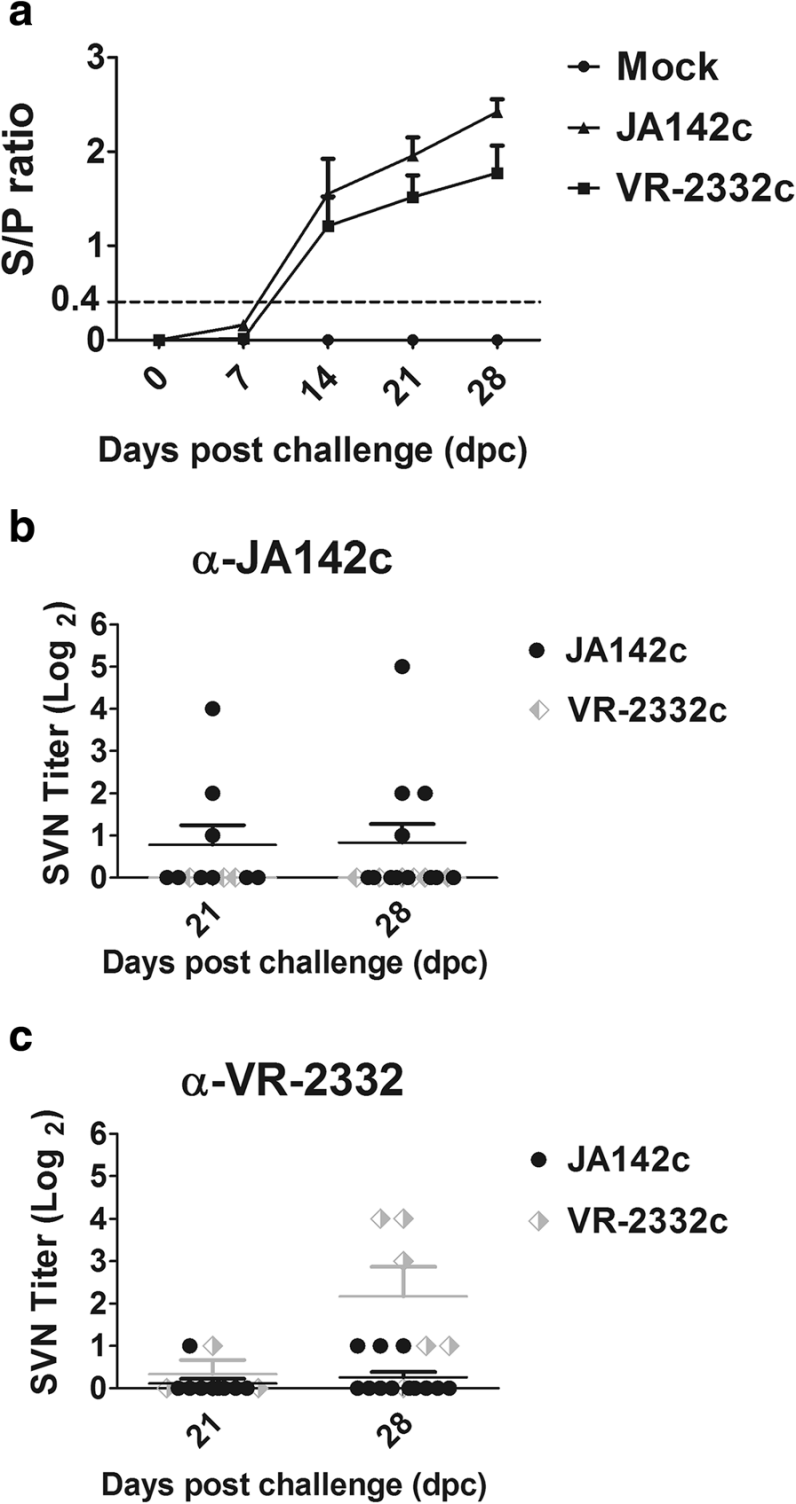
Antibody and Nab titres in PRRSV-infected pigs. **a** Weekly antibody titres in infected pigs were measured by performing a PRRSV ELISA. The 0.4 S/P ratio represents the designated threshold value and is indicated by a dashed line. **b** SVN antibody titres of sera collected from pigs infected with JA142c (*n* = 5) or VR2332c (*n* = 5) tested against JA142c at 21 and 28 dpc. **c** SVN antibody titres of sera collected from pigs infected with JA142c or VR2332c tested against VR2332c at 21 and 28 dpc

The PRRSV-specific antibody (IgG) response was measured by performing a nucleocapsid (N) protein-based ELISA for the two virus-challenged groups. The pigs seroconverted after 2 weeks of exposure to PRRSV and remained seropositive until the end of the study (Fig. 8a). The negative control group remained seronegative until the end of the study. No significant differences in the IgG response were detected between the JA142c- and VR2332c-challenged groups.

NAb levels in the collected serum samples were determined using an FFN assay. At 28 dpc, only homologous NAbs were induced, and no significant cross-NAbs were induced by either VR2332c or JA142c (Fig. 8b and c).

### Lung pathology and viral load

**Fig. 9:**
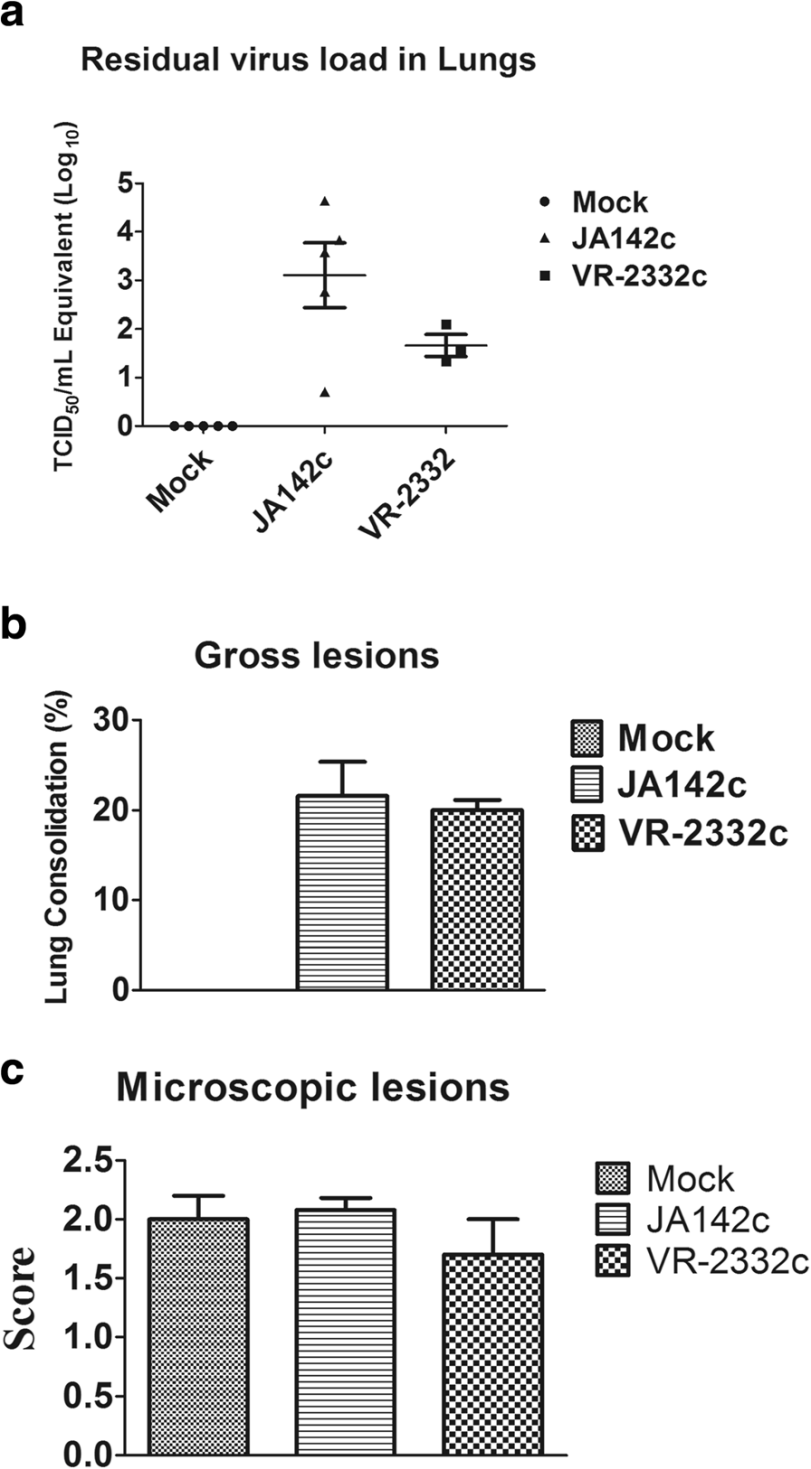
Quantification of residual viral loads in the lungs and evaluation of gross and microscopic lung lesions at 28 dpc. **a** Residual viral loads in the lungs of PRRSV-infected pigs at 28 dpc. Viral titres were calculated based on the standard curve of the cycle threshold (Ct) number plotted against the known viral titre of VR2332c. **b** Gross and **c** microscopic lung scores were recorded after necropsy. Lung scores were plotted as the mean values of the lesion scores from 5 pigs in each group, and the error bars represent the SEM

JA142c-infected pigs (3.1 ± 0.67 TCID_50_/mL) had higher residual viral loads in the lung than VR2332c-infected pigs (1.662 ± 0.22 TCID_50_/mL); however, this difference was not statistically significant (Fig. 9a). Furthermore, there were no significant differences in gross or microscopic lung lesion scores among the groups challenged with JA142c or VR2332c (Fig. 9b & c).

## Discussion

It has been demonstrated that type I IFNs (IFN-α and IFN-β) play an important role in the induction of innate and adaptive immunity during PRRSV infection [42, 43]. In the current study, JA142c induced higher IFN-α production in PAMs than did VR2332c. This differential IFN-α induction in PAMs is consistent with the results of previous study that demonstrated variable IFN-α production in response to different Type II PRRSV isolates [22]. Moreover, compared with VR2332c, JA142c induced the production of significantly increased serum IFN-α levels in pigs from 3 to 14 dpc, which is in partial agreement with a previous study demonstrating lower levels of serum IFN-α in VR2332c-infected pigs [44]. In addition, JA142c induced significantly higher IFN-β transcription than did VR2332c in naïve PAMs, similar to the results of previous studies describing high IFN-β transcription in PAMs following infection by several PRRSV strains [45–47]. Because PRRSV could modulate and block type I IFN induction [39], these results indicated that VR2332c might exert enhanced inhibitory effects on type I IFN production in PAMs as well as in pigs. Furthermore, in the present study, JA142c induced significantly higher TNF-α production in PAMs and pigs than did VR2332c. This observation is consistent with previous studies that reported various levels of TNF-α induction in PRRSV-infected PAMs, PBMCs, or pigs [9, 19, 20, 25, 44, 48–50]. TNF-α is another major pro-inflammatory cytokine that promotes the antiviral state in uninfected neighbouring cells and recruits lymphocytes to sites of infection [48]. Similarly, significantly higher mRNA transcription of other pro-inflammatory cytokines (IL-1α/β, IL-6, IL-8 and IL-12) was also observed in PAMs and JA142c-challenged pigs at 7 dpc than in VR2332c-challenged pigs, which is partially consistent with previous studies that demonstrated lower levels of IL-1β, IL-6 and IL-8 in VR2332c-challenged pigs [44, 51]. Therefore, it was concluded that compared with VR2332c, JA142c consistently induced significantly higher levels of important pro-inflammatory cytokines in PAMs and pigs.

When T cell responses were analysed in pigs challenged with JA142c and VR2332c, compared with those from VR2332c- or mock-challenged pigs, PBMCs from JA142c-challenged pigs contained significantly larger populations of activated CD4^+^ T cells (CD4^+^CD25^+^) and exhibited significant and early increases in type I helper T cells (CD4^+^IFN-γ^+^) and total IFN-γ^+^ cells. Cytotoxic T cells (CD8^+^IFN-γ^+^) in both JA142c- and VR2332c-infected pigs increased at 14 dpc, but the JA142c group had a significantly larger population of cytotoxic T lymphocytes (CTLs) than the mock- and VR2332c-infected groups. In a previous study, low numbers of PRRSV-specific IFN-γ^+^ CD8^+^ T cells were associated with a delayed CTL response [33].

## Conclusions

Collectively, based on the results of the current study, JA142c induced more efficient innate and adaptive immune responses in vitro and in vivo than did VR2332c. Furthermore, of the two in vitro screening systems for immunogenicity (PAMs and PBMCs), PAMs generated immune response patterns to both PRRSVs that were similar to those elicited by these viruses in pigs. Thus, PAMs may be used as an immunogenicity screening tool for the selection of vaccine candidates against PRRSV. Finally, the induction of IFN-α, TNF-α and IL-12 in PAMs might represent vital immune markers to predict the immunogenicity of PRRSV vaccine candidates in pigs.

## Methods

### Cells and viruses

MARC-145, an African green monkey kidney cell line that is highly permissive to PRRSV, was used for virus propagation and maintained in Roswell Park Memorial Institute (RPMI)-1640 medium (Gibco® RPMI 1640, Life Technologies, Carlsbad, CA, USA) supplemented with heat-inactivated 10% fetal bovine serum (FBS, Life Technologies), 2 mM L-glutamine, and 100X Antibiotic-Antimycotic (Anti-anti, Life Technologies) containing 100 IU/mL penicillin, 100 μg/mL streptomycin, and 0.25 μg/mL Fungizone® (amphotericin B) in a 5% CO_2_ humidified chamber at 37 °C. In this study, this medium is referred to as complete RPMI (cRPMI) medium. Two infectious clones of Type II PRRSV strains, JA142 and VR2332 [52, 53], were re-cloned into a vector (pOptiVEC™-TOPO® TA Cloning Kit, Life Technologies) to construct modified infectious clones by inserting viral sequences between the human cytomegalovirus (CMV) promoter and the internal ribosomal entry site (IRES) that are present in the vector [54, 55]. The viruses rescued from each of the modified infectious clones were named JA142c and VR2332c. The infectious clone-driven viruses were used in this study to minimize mutations caused by passaging the virus in cells.

### Harvesting porcine alveolar macrophages (PAMs) and collecting peripheral blood mononuclear cells (PBMCs)

PAMs were harvested from six 6-week-old PRRSV-negative pigs, as described previously [56]. The pigs were euthanized, and the lungs, trachea and bronchus were aseptically collected. The lungs were lavaged three times with 50 mL of phosphate-buffered saline (PBS, pH 7.4), and the harvested wash fluid was centrifuged for 10 min at 1000 *g*. The resulting pellet was washed three times with PBS and re-suspended in 5 mL of sterile PBS. To evaluate cell number and viability, the cells were diluted 100-fold in PBS, mixed with 0.4% trypan blue at a 1:1 ratio and counted using a Countess™ Automated Cell Counter (Invitrogen, Carlsbad, CA, USA). After counting, freezing medium (70% Dulbecco’s modified Eagle’s medium (DMEM) with high glucose, pyruvate, 20% FBS [Gibco, California, USA], and 10% dimethyl sulfoxide [Sigma-Aldrich, St. Louis, Missouri, USA]) was added to the cells to maintain the final cell density at 5 × 10^7^ cells/mL. The cells were collected in cryovials and stored at − 80 °C until use.

PBMCs were isolated from 6-mL blood samples collected from six-week-old PRRSV-free pigs in lithium-heparin-containing vacutainers using a density gradient method in Histopaque-1077® solution (Sigma, St. Louis, MO, USA) according to the manufacturer’s instructions. The blood samples were briefly stratified in Histopaque-1077® solution at a ratio of 1:1 (blood:Histopaque) and were centrifuged at 400 *g* for 30 min. Purified PBMCs were collected, washed twice with sterile PBS (pH 7.0) supplemented with 1% FBS (Gibco, Carlsbad, CA, USA) and re-suspended in 0.5 mL of sterile PBS.

### In vitro evaluation of the growth kinetics of JA142c and VR2332c

The growth of JA142c and VR2332c was evaluated in MARC-145 cells. Confluent monolayers of MARC-145 cells were prepared in nine 25 cm^2^ flasks, with three flasks inoculated with each virus or mock inoculated at a multiplicity of infection (MOI) of 0.01 and incubated for 1 h in a humidified chamber at 37 °C. After incubation, the cells were replenished with cRPMI and incubated for an additional 48 h. Supernatants were collected from each flask at 6, 12, 24, 36 and 48 h, and the virus in these supernatants was titrated as described previously [57].

### Animal study

A total of 15 three-week-old PRRSV-free pigs were purchased from a farm which has been PRRS-negative over last 10 years and randomly divided into 3 groups and housed separately in the animal research facility in Chonbuk National University. Five pigs in each group were challenged with VR2332c or JA142c diluted in RPMI to 10^3^ TCID_50_/mL in a volume of 2 mL or with mock inoculum (sterile PBS) injected intramuscularly and housed for 4 weeks. All pigs were bled at 0, 3, 7, 14, 21 and 28 days post-challenge (dpc), and whole blood was collected for PBMC isolation to evaluate cytokine mRNA transcript levels and perform flow cytometry. Sera were separated immediately after bleeding and stored at − 80 °C until use. Viremia and serum cytokine levels in all pigs were evaluated weekly, at which point all pigs were weighed. Serum virus neutralizing antibody induction was evaluated at 21 and 28 dpc. All pigs were humanly euthanized at 28 dpc by electrocution after intramuscular injection of 3 ml of Azaperone (40 mg/ml, StressGuar®, Dong Bang Inc., Seoul, South Korea) for sedation and subjected to pathological evaluation. To evaluate gross and microscopic lung lesions, each lung lobe was scored for the percentage of lung consolidation [58] and interstitial pneumonia resulting from PRRSV infection. Scoring of microscopic lung lesions was recorded as follows: 0 indicates no lesion; 1 indicates mild interstitial pneumonia; 2 indicates moderate multifocal interstitial pneumonia; 3 indicates moderate diffuse interstitial pneumonia; and 4 indicates severe interstitial pneumonia. Lung tissues were collected from each pig and stored at − 80 °C until examination.

### Transcriptional activation of cellular cytokines

PAMs re-suspended in cRPMI at 5 × 10^6^ cells/mL were seeded in each well of a 24-well plate (BD Falcon, Franklin Lakes, NJ, USA) and cultured overnight in a 5% CO_2_ humidified chamber at 37 °C. The cultured PAMs were stimulated with a 0.1 MOI of either JA142c or VR2332c, 10 μg/mL polyinosinic-polycytidylic acid (poly I:C) (Sigma-Aldrich, St. Louis, MO, USA) or mock inoculum (cRPMI) and incubated in a 5% CO_2_ humidified chamber at 37 °C. Six PAMs harvested from six PRRSV-free pigs were used in the experiment. The cells were harvested, and the cell lysates were collected at 12, 24 and 36 hpi. Similarly, 1 × 10^6^ naïve PBMCs re-suspended in cRPMI were seeded in each well of a 24-well plate. The cells were stimulated with a 0.1 MOI of either JA142c or VR2332c, 10 μg/mL poly I:C or mock inoculum (cRPMI) and incubated in a 5% CO_2_ humidified chamber at 37 °C, after which the cells were harvested, and the cell supernatants were collected at 12, 36 and 72 hpi. Four PBMC cells from 4 PRRSV-free pigs were used for the experiment. Poly I:C, a synthetic analogue of double-stranded RNA, was used as an immune-inducing positive control for PAMs and PBMCs. Cellular RNA was extracted using an RNA isolation kit (GeneAll® Hybrid-RTM kit, GeneAll Biotechnology, Seoul, South Korea) following the manufacturer’s instructions. RNA was analysed on agarose gels as initial quality checks and 260/280 and 260/230 ratios were determined to be 1.8–2.2 and 1.7 or higher, respectively. Then, 1 μg of RNA was used for complementary DNA (cDNA) synthesis using a high-capacity cDNA reverse transcription kit (Applied Biosystems, Foster City, CA, USA) following the manufacturer’s instructions. Real-time PCR was then performed on a 7500 Fast Real-time PCR system (Applied Biosystems) using various cytokine-specific primers, following the manufacturer’s instructions. The primer sequences used in this study are shown in Table 1. Ten microlitres of 2X Power SYBR Green PCR Master Mix (Applied Biosystems, Foster City, CA, USA), 2 μL of cDNA and 1 μL of each forward and reverse primer (each at 10 pm/μL) were used for PCR amplification. All samples were tested in duplicate, and the following cycling conditions were applied: (a) incubation for 10 min at 95 °C; (b) 40 cycles of 15 s at 95 °C and 1 min at 60 °C; and (c) a melting curve stage of 15 s at 95 °C, 1 min at 60 °C, 15 s at 95 °C and 15 s at 60 °C. The relative quantities of cytokine mRNA in infected and non-infected cells were normalized to β-actin mRNA, and the amounts were determined using the 2^-ΔΔCt^ method [59].

**Table 1:** Information about the real-time PCR primers used to measure mRNA transcript levels of various cytokines

Genes	Forward Primer (5′- 3′)	Reverse Primer (5′- 3′)	Accession/Reference
β-Actin	GCGGGACATCAAGGAGAAG	AGGAAGGAGGGCTGGAAGAG	U07786
IFN-α	TCTCATGCACCAGAGCCA	CCTGGACCACAGAAGGGA	[39]
IFN-β	AGTGCATCCTCCAAATCGCT	GCTCATGGAAAGAGCTGTGGT	M86762
TNF-α	TTATTCAGGAGGGCGAGGT	AGCAAAAGGAGGCACAGAGG	NM214022
IL-1α	GTGCTCAAAACGAAGACGAACC	CATATTGCCATGCTTTTCCCAGAA	NM_214029.1
IL-1β	AACGTGCAGTCTATGGAGT	GAACACCACTTCTCTCTTCA	M86725
IL-6	CCACCAGGAACGAAAGAGAG	AGGCAGTAGCCATCACCAGA	NM_214399
IL-8	TAGGACCAGAGCCAGGAAGA	CAGGAAAACTGCCAAGAAGG	M86923.1
IL-10	TGACGATGAAGATGAGGAAGAA	GAACCTTGGAGCAGATTTTGA	NM214041
IL-12	TCAGGGACATCATCAAACCA	GAACACCAAACATCAGGGAAA	NM214013
IFN-γ	GACTTTGTGTTTTTCTGGCTCTTAC	TTTTGTCACTCTCCTCTTTCCA	NM213948
IL-2	TGCACTAACCCTTGCACTCA	CCTGCTTGGGCATGTAAAAT	X56750
IL-4	TTTGCTGCCCCAGAGAAC	TCCTGTCAAGTCCGCTCA	X68330

*IL* interleukin, *IFN* interferon

### Cytokine quantification by ELISA

Commercially available porcine-specific ELISA kits were used to quantify various cytokine protein levels in sera, cell supernatants or lysates, including TNF-α, IL-4, IL-10, and IL-12 (DuoSet® ELISA, R&D Systems, MN, USA), according to the manufacturer’s instructions.

To detect IFN-α protein levels in sera and cell lysates, 100 μL (1.8 μg/mL) of a mouse anti-pig IFN-α antibody (Clone F17, PBL Assay Science, NJ, USA) was used as a coating antibody, and a mouse anti-pig IFN-α antibody (Clone K9, PBL Assay Science, NJ, USA) was biotinylated and used as a secondary antibody, with recombinant porcine IFN-α (PBL Assay Science, NJ, USA) as a standard. The procedure was carried out using the provided ELISA reagents (eBioscience, CA, USA) and following the manufacturer’s instructions. The results were analysed using SoftMax Pro 5.3 microplate data software (Molecular Devices, CA, USA).

### Type I IFN bioassay

A conventional type I IFN bioassay using sera from virus-challenged pigs was performed to assess the amount of virus-induced type I IFNs present by determining anti-vesicular stomatitis virus (VSV) activity. MDBK cells were seeded in 96-well cell culture plates (BD Falcon, Franklin Lakes, NJ, USA) (5 × 10^5^ cells/well) and incubated for 20 h in the presence of two-fold dilutions of recombinant IFN-α (r-IFN-α) (PBL Assay Science, NJ, USA), as described previously. The cells were infected with propagation-competent VSV (0.01 MOI) and incubated until a cytopathic effect (CPE) was evident in mock-treated control cells. The cells were washed twice with PBS and stained for 1 h with 0.1% crystal violet in 10% formalin. The plates were then washed with tap water to remove excess crystal violet and dried. The dye was dissolved by adding 100 mL of 70% ethanol to each well. Crystal violet absorbance at 595 nm was determined with a microplate reader. Antiviral activity was calculated as described previously [54].

### Quantification of PRRSV RNA in sera and lungs

Viral RNA was extracted from 100 μL of serum and 1 g of lung samples using a viral RNA extraction kit (MagMAX™ Viral RNA Isolation Kit, Life Technologies) and a total RNA extraction kit (Hybrid-RTM, GeneAll, Seoul, Korea) according to the manufacturer’s instructions. The viral loads in sera and lungs were measured by performing real-time RT-PCR employing a one-step reverse transcriptase kit (AgPath-IDTM One-Step RT-PCR Kit, Ambion, Austin, TX, USA) with a 7500 Fast Real-time PCR system (Applied Biosystems, Foster City, CA, USA), although this test cannot differentiate between replication-competent and replication-incompetent virus. Primers and a minor groove binder (MGB) fluorescent probe specific to a conserved region of ORF7 were employed as described previously [60].

### Assessment of PRRSV-specific antibodies

A fluorescence focus neutralization (FFN)-based serum virus neutralization (SVN) assay was performed to evaluate NAb titres induced by PRRSVs after challenge. The SVN assay was performed in MARC-145 cells as described previously [61, 62]. The NAb titres of each anti-serum against each virus were expressed as the reciprocal of the highest dilution for which a 90% or greater reduction in the number of fluorescent focus units (FFU)/mL was observed compared to the wells for each respective virus back-titration.

The presence of PRRSV nucleocapsid-specific antibodies in the sera of infected animals was determined using a direct ELISA kit (HerdCheck® PRRS Antibody Kit 3XR, IDEXX Laboratories, Westbrook, ME, USA) according to the manufacturer’s instructions.

### Flow cytometry

The frequency and phenotype of the immune cell populations were determined by performing multicolour immunostaining of single-cell suspensions on the day of PBMC isolation. PBMCs were evaluated for the expression of markers of activated CD4^+^ T cells (CD4^+^CD25^+^FoxP3^−^), regulatory T cells (CD4^+^CD25^+^FoxP3^+^), type I helper T cells (CD4^+^IFN-γ^+^), and cytotoxic T cells (CD8^+^IFN-γ^+^). The cells were stained with an appropriate monoclonal antibody (mAb), which was either directly conjugated to a specific fluorochrome or biotinylated, or with a purified antibody targeting porcine-specific immune cell surface markers as described previously [63]. Briefly, 1 × 10^6^ purified PBMCs per sample were re-suspended in fluorescence-activated cell sorting (FACS) buffer (2% FBS in PBS with 0.02% sodium azide), and two replicates of each sample were plated in two separate U-bottom 96-well plates with 1 × 10^6^ cells per well in a 200-μL volume. Subsequently, the cells in one plate were stained with either CD4α-PE (Clone 74–12-4; BD Biosciences, Franklin Lakes, New Jersey, USA) or anti-porcine CD25 (clone K231.3B3; Serotech, Raleigh, NC), followed by washing and staining with an allophycocyanin-conjugated rat anti-mouse IgG1 antibody (Ab) (Clone RMG1–1; Biolegend, San Diego, CA, USA) as a secondary antibody against anti-CD25 according to the manufacturer’s instructions. Afterwards, the cells were fixed and stained with a FoxP3-FITC antibody (Clone FJK-16 s; eBiosciences, San Diego, CA, USA), as described. Simultaneously, two other sets of cells were treated with a 1X cell stimulation cocktail (eBiosciences) plus 1X brefeldin A (eBiosciences) in cRPMI media and incubated at 37 °C in a 5% CO_2_ humidified chamber for 5 h. Afterwards, one set of cells was stained with CD8α-FITC (Clone 76–2-11; BD Biosciences), and the other set of cells was stained with CD4α-PE and fixed with IC fixation buffer (eBiosciences) according to the manufacturer’s instructions. Finally, the cells were stained with IFN-γ-PerCP-Cy™ 5.5 (Clone P2G10; BD Biosciences). A total of 100,000 events (gated by forward and side scatter) were acquired for each sample using an Accuri C6 flow cytometer (BD Biosciences, Franklin Lakes, New Jersey, USA), and the data were analysed using BD CFlow®Plus software v. 1.0.227.4 (BD Biosciences, Franklin Lakes, New Jersey, USA). Target cell frequencies were expressed as the percentage for a specific cell subset.

### Data analysis

Graphs were constructed using GraphPad Prism 5.0.2 (GraphPad, San Diego, CA, USA), and statistical analyses were performed using SPSS Advanced Statistics 17.0 software (SPSS, Inc., Chicago, USA). The Mann-Whitney *U* test was applied to estimate differences between different groups. Repeated measurements of viremia and PRRSV nucleocapsid-specific antibody levels in challenged pigs were analysed using a repeated ANOVA test (Tukey’s post hoc test) to determine the overall difference, and pairwise comparisons were also made between groups.

## Abbreviations

CTLs: Cytotoxic T lymphocytes
IFNs: Interferons
Nabs: Neutralizing antibodies
NSPs: Non-structural proteins
PAMs: Porcine alveolar macrophages
PBMCs: Peripheral blood mononuclear cells
PRRS: Porcine reproductive and respiratory syndrome
T_Regs_: Regulatory T cells

## References

[1] Cavanagh D (1997). Nidovirales: a new order comprising Coronaviridae and Arteriviridae. Arch Virol.

[2] Collins JE, Benfield DA, Christianson WT, Harris L, Hennings JC, Shaw DP, Goyal SM, McCullough S, Morrison RB, Joo HS (1992). Isolation of swine infertility and respiratory syndrome virus (isolate ATCC VR-2332) in North America and experimental reproduction of the disease in gnotobiotic pigs. J Vet Diagn Investig.

[3] Wensvoort G, Terpstra C, Pol JM, ter Laak EA, Bloemraad M, de Kluyver EP, Kragten C, van Buiten L, den Besten A, Wagenaar F (1991). Mystery swine disease in the Netherlands: the isolation of Lelystad virus. Vet Q.

[4] Holtkamp DJ, Kliebenstein JB, Neumann E, Zimmerman JJ, Rotto H, Yoder TK, Wang C, Yeske P, Mowrer CL, Haley CA (2013). Assessment of the economic impact of porcine reproductive and respiratory syndrome virus on United States pork producers. J Swine Health Prod.

[5] Firth AE, Zevenhoven-Dobbe JC, Wills NM, Go YY, Balasuriya UB, Atkins JF, Snijder EJ, Posthuma CC (2011). Discovery of a small arterivirus gene that overlaps the GP5 coding sequence and is important for virus production. J Gen Virol.

[6] Johnson CR, Griggs TF, Gnanandarajah J, Murtaugh MP (2011). Novel structural protein in porcine reproductive and respiratory syndrome virus encoded by an alternative ORF5 present in all arteriviruses. J Gen Virol.

[7] Shi M, Lam TT, Hon CC, Hui RK, Faaberg KS, Wennblom T, Murtaugh MP, Stadejek T, Leung FC (2010). Molecular epidemiology of PRRSV: a phylogenetic perspective. Virus Res.

[8] Shi M, Lam TT, Hon CC, Murtaugh MP, Davies PR, Hui RK, Li J, Wong LT, Yip CW, Jiang JW (2010). Phylogeny-based evolutionary, demographical, and geographical dissection of North American type 2 porcine reproductive and respiratory syndrome viruses. J Virol.

[9] Gimeno M, Darwich L, Diaz I, de la Torre E, Pujols J, Martin M, Inumaru S, Cano E, Domingo M, Montoya M (2011). Cytokine profiles and phenotype regulation of antigen presenting cells by genotype-I porcine reproductive and respiratory syndrome virus isolates. Vet Res.

[10] Silva-Campa E, Cordoba L, Fraile L, Flores-Mendoza L, Montoya M, Hernandez J (2010). European genotype of porcine reproductive and respiratory syndrome (PRRSV) infects monocyte-derived dendritic cells but does not induce Treg cells. Virology.

[11] Meng XJ (2000). Heterogeneity of porcine reproductive and respiratory syndrome virus: implications for current vaccine efficacy and future vaccine development. Vet Microbiol.

[12] Vu Hiep L.X., Pattnaik Asit K., Osorio Fernando A. (2017). Strategies to broaden the cross-protective efficacy of vaccines against porcine reproductive and respiratory syndrome virus. Veterinary Microbiology.

[13] Park JY, Kim HS, Seo SH (2008). Characterization of interaction between porcine reproductive and respiratory syndrome virus and porcine dendritic cells. J Microbiol Biotechnol.

[14] Song S, Bi J, Wang D, Fang L, Zhang L, Li F, Chen H, Xiao S (2013). Porcine reproductive and respiratory syndrome virus infection activates IL-10 production through NF-kappaB and p38 MAPK pathways in porcine alveolar macrophages. Dev Comp Immunol.

[15] Wang G, Song T, Yu Y, Liu Y, Shi W, Wang S, Rong F, Dong J, Liu H, Cai X (2011). Immune responses in piglets infected with highly pathogenic porcine reproductive and respiratory syndrome virus. Vet Immunol Immunopathol.

[16] Albina E, Carrat C, Charley B (1998). Interferon-alpha response to swine arterivirus (PoAV), the porcine reproductive and respiratory syndrome virus. J Interferon Cytokine Res.

[17] Buddaert W, Van Reeth K, Pensaert M (1998). In vivo and in vitro interferon (IFN) studies with the porcine reproductive and respiratory syndrome virus (PRRSV). Adv Exp Med Biol.

[18] Han M, Yoo D (2014). Modulation of innate immune signaling by nonstructural protein 1 (nsp1) in the family Arteriviridae. Virus Res.

[19] Gomez-Laguna J, Salguero FJ, Pallares FJ, Carrasco L (2013). Immunopathogenesis of porcine reproductive and respiratory syndrome in the respiratory tract of pigs. Vet J.

[20] Lopez-Fuertes L, Campos E, Domenech N, Ezquerra A, Castro JM, Dominguez J, Alonso F (2000). Porcine reproductive and respiratory syndrome (PRRS) virus down-modulates TNF-alpha production in infected macrophages. Virus Res.

[21] Baumann A, Mateu E, Murtaugh MP, Summerfield A (2013). Impact of genotype 1 and 2 of porcine reproductive and respiratory syndrome viruses on interferon-alpha responses by plasmacytoid dendritic cells. Vet Res.

[22] Lee SM, Schommer SK, Kleiboeker SB (2004). Porcine reproductive and respiratory syndrome virus field isolates differ in in vitro interferon phenotypes. Vet Immunol Immunopathol.

[23] He Q, Li Y, Zhou L, Ge X, Guo X, Yang H (2015). Both Nsp1beta and Nsp11 are responsible for differential TNF-alpha production induced by porcine reproductive and respiratory syndrome virus strains with different pathogenicity in vitro. Virus Res.

[24] Diaz I, Darwich L, Pappaterra G, Pujols J, Mateu E (2006). Different European-type vaccines against porcine reproductive and respiratory syndrome virus have different immunological properties and confer different protection to pigs. Virology.

[25] Aasted B, Bach P, Nielsen J, Lind P (2002). Cytokine profiles in peripheral blood mononuclear cells and lymph node cells from piglets infected in utero with porcine reproductive and respiratory syndrome virus. Clin Diagn Lab Immunol.

[26] Thanawongnuwech R, Young TF, Thacker BJ, Thacker EL (2001). Differential production of proinflammatory cytokines: in vitro PRRSV and mycoplasma hyopneumoniae co-infection model. Vet Immunol Immunopathol.

[27] Liu CH, Chaung HC, Chang HL, Peng YT, Chung WB (2009). Expression of toll-like receptor mRNA and cytokines in pigs infected with porcine reproductive and respiratory syndrome virus. Vet Microbiol.

[28] Peng YT, Chaung HC, Chang HL, Chang HC, Chung WB (2009). Modulations of phenotype and cytokine expression of porcine bone marrow-derived dendritic cells by porcine reproductive and respiratory syndrome virus. Vet Microbiol.

[29] van Gucht S, van Reeth K, Pensaert M (2003). Interaction between porcine reproductive-respiratory syndrome virus and bacterial endotoxin in the lungs of pigs: potentiation of cytokine production and respiratory disease. J Clin Microbiol.

[30] Meier WA, Husmann RJ, Schnitzlein WM, Osorio FA, Lunney JK, Zuckermann FA (2004). Cytokines and synthetic double-stranded RNA augment the T helper 1 immune response of swine to porcine reproductive and respiratory syndrome virus. Vet Immunol Immunopathol.

[31] Xiao Z, Trincado CA, Murtaugh MP (2004). Beta-glucan enhancement of T cell IFNgamma response in swine. Vet Immunol Immunopathol.

[32] Costers S, Lefebvre DJ, Goddeeris B, Delputte PL, Nauwynck HJ (2009). Functional impairment of PRRSV-specific peripheral CD3+CD8high cells. Vet Res.

[33] Ferrari L, Martelli P, Saleri R, De Angelis E, Cavalli V, Bresaola M, Benetti M, Borghetti P (2013). Lymphocyte activation as cytokine gene expression and secretion is related to the porcine reproductive and respiratory syndrome virus (PRRSV) isolate after in vitro homologous and heterologous recall of peripheral blood mononuclear cells (PBMC) from pigs vaccinated and exposed to natural infection. Vet Immunol Immunopathol.

[34] Silva-Campa E, Mata-Haro V, Mateu E, Hernandez J (2012). Porcine reproductive and respiratory syndrome virus induces CD4+CD8+CD25+Foxp3+ regulatory T cells (Tregs). Virology.

[35] Rodriguez-Gomez IM, Kaser T, Gomez-Laguna J, Lamp B, Sinn L, Rumenapf T, Carrasco L, Saalmuller A, Gerner W (2015). PRRSV-infected monocyte-derived dendritic cells express high levels of SLA-DR and CD80/86 but do not stimulate PRRSV-naive regulatory T cells to proliferate. Vet Res.

[36] Darwich L, Diaz I, Mateu E (2010). Certainties, doubts and hypotheses in porcine reproductive and respiratory syndrome virus immunobiology. Virus Res.

[37] Kimman TG, Cornelissen LA, Moormann RJ, Rebel JM, Stockhofe-Zurwieden N (2009). Challenges for porcine reproductive and respiratory syndrome virus (PRRSV) vaccinology. Vaccine.

[38] Mateu E, Diaz I (2008). The challenge of PRRS immunology. Vet J.

[39] Loving CL, Osorio FA, Murtaugh MP, Zuckermann FA (2015). Innate and adaptive immunity against porcine reproductive and respiratory syndrome virus. Vet Immunol Immunopathol.

[40] Plagemann PG (2006). Neutralizing antibody formation in swine infected with seven strains of porcine reproductive and respiratory syndrome virus as measured by indirect ELISA with peptides containing the GP5 neutralization epitope. Viral Immunol.

[41] Kim WI, Lee DS, Johnson W, Roof M, Cha SH, Yoon KJ (2007). Effect of genotypic and biotypic differences among PRRS viruses on the serologic assessment of pigs for virus infection. Vet Microbiol.

[42] Brockmeier SL, Loving CL, Nelson EA, Miller LC, Nicholson TL, Register KB, Grubman MJ, Brough DE, Kehrli ME (2012). The presence of alpha interferon at the time of infection alters the innate and adaptive immune responses to porcine reproductive and respiratory syndrome virus. Clin Vaccine Immunol.

[43] Royaee AR, Husmann RJ, Dawson HD, Calzada-Nova G, Schnitzlein WM, Zuckermann FA, Lunney JK (2004). Deciphering the involvement of innate immune factors in the development of the host response to PRRSV vaccination. Vet Immunol Immunopathol.

[44] Guo B, Lager KM, Henningson JN, Miller LC, Schlink SN, Kappes MA, Kehrli ME, Brockmeier SL, Nicholson TL, Yang HC (2013). Experimental infection of United States swine with a Chinese highly pathogenic strain of porcine reproductive and respiratory syndrome virus. Virology.

[45] Gudmundsdottir I, Risatti GR (2009). Infection of porcine alveolar macrophages with recombinant chimeric porcine reproductive and respiratory syndrome virus: effects on cellular gene transcription and virus growth. Virus Res.

[46] Badaoui B, Rutigliano T, Anselmo A, Vanhee M, Nauwynck H, Giuffra E, Botti S (2014). RNA-sequence analysis of primary alveolar macrophages after in vitro infection with porcine reproductive and respiratory syndrome virus strains of differing virulence. PLoS One.

[47] Overend CC, Cui J, Grubman MJ, Garmendia AE (2017). The activation of the IFNbeta induction/signaling pathway in porcine alveolar macrophages by porcine reproductive and respiratory syndrome virus is variable. Vet Res Commun.

[48] Huang Chen, Zhang Qiong, Feng Wen-hai (2015). Regulation and evasion of antiviral immune responses by porcine reproductive and respiratory syndrome virus. Virus Research.

[49] Ait-Ali T, Wilson AD, Westcott DG, Clapperton M, Waterfall M, Mellencamp MA, Drew TW, Bishop SC, Archibald AL (2007). Innate immune responses to replication of porcine reproductive and respiratory syndrome virus in isolated swine alveolar macrophages. Viral Immunol.

[50] Choi C, Cho WS, Kim B, Chae C (2002). Expression of interferon-gamma and tumour necrosis factor-alpha in pigs experimentally infected with porcine reproductive and respiratory syndrome virus (PRRSV). J Comp Pathol.

[51] Manickam C, Dwivedi V, Patterson R, Papenfuss T, Renukaradhya GJ (2013). Porcine reproductive and respiratory syndrome virus induces pronounced immune modulatory responses at mucosal tissues in the parental vaccine strain VR2332 infected pigs. Vet Microbiol.

[52] Truong HM, Lu Z, Kutish GF, Galeota J, Osorio FA, Pattnaik AK (2004). A highly pathogenic porcine reproductive and respiratory syndrome virus generated from an infectious cDNA clone retains the in vivo virulence and transmissibility properties of the parental virus. Virology.

[53] Nielsen HS, Liu G, Nielsen J, Oleksiewicz MB, Botner A, Storgaard T, Faaberg KS (2003). Generation of an infectious clone of VR-2332, a highly virulent North American-type isolate of porcine reproductive and respiratory syndrome virus. J Virol.

[54] Berger Rentsch M, Zimmer G (2011). A vesicular stomatitis virus replicon-based bioassay for the rapid and sensitive determination of multi-species type I interferon. PLoS One.

[55] Sun D, Khatun A, Kim WI, Cooper V, Cho YI, Wang C, Choi EJ, Yoon KJ (2016). Attempts to enhance cross-protection against porcine reproductive and respiratory syndrome viruses using chimeric viruses containing structural genes from two antigenically distinct strains. Vaccine.

[56] OIE (2011). Porcine reproductive and respiratory syndrome.

[57] Khatun A, Shabir N, Yoon KJ, Kim WI (2015). Effects of ribavirin on the replication and genetic stability of porcine reproductive and respiratory syndrome virus. BMC Vet Res.

[58] Halbur PG, Miller LD, Paul PS, Meng XJ, Huffman EL, Andrews JJ (1995). Immunohistochemical identification of porcine reproductive and respiratory syndrome virus (PRRSV) antigen in the heart and lymphoid system of three-week-old colostrum-deprived pigs. Vet Pathol.

[59] Winer J, Jung CK, Shackel I, Williams PM (1999). Development and validation of real-time quantitative reverse transcriptase-polymerase chain reaction for monitoring gene expression in cardiac myocytes in vitro. Anal Biochem.

[60] Opriessnig T, McKeown NE, Harmon KL, Meng XJ, Halbur PG (2006). Porcine circovirus type 2 infection decreases the efficacy of a modified live porcine reproductive and respiratory syndrome virus vaccine. Clin Vaccine Immunol.

[61] Kim WI, Kim JJ, Cha SH, Yoon KJ (2008). Different biological characteristics of wild-type porcine reproductive and respiratory syndrome viruses and vaccine viruses and identification of the corresponding genetic determinants. J Clin Microbiol.

[62] Wu WH, Fang Y, Farwell R, Steffen-Bien M, Rowland RR, Christopher-Hennings J, Nelson EA (2001). A 10-kDa structural protein of porcine reproductive and respiratory syndrome virus encoded by ORF2b. Virology.

[63] Niu P, Shabir N, Khatun A, Seo BJ, Gu S, Lee SM, Lim SK, Kim KS, Kim WI (2016). Effect of polymorphisms in the GBP1, Mx1 and CD163 genes on host responses to PRRSV infection in pigs. Vet Microbiol.

